# The Association between School Corporal Punishment and Child Developmental Outcomes: A Meta-Analytic Review

**DOI:** 10.3390/children9030383

**Published:** 2022-03-09

**Authors:** Lotte N. Visser, Claudia E. van der Put, Mark Assink

**Affiliations:** Research Institute of Child Development and Education, University of Amsterdam, 1018 WS Amsterdam, The Netherlands; lotte.visser@student.uva.nl (L.N.V.); c.e.vanderput@uva.nl (C.E.v.d.P.)

**Keywords:** school corporal punishment, externalizing behavior, internalizing behavior, school performance, child development, meta-analysis

## Abstract

School corporal punishment (SCP) is still widely used in many countries. Although primary studies have pointed toward detrimental effects of SCP, a quantitative review of these studies was not yet available. To gain better insight into effects of SCP, three meta-analyses were conducted on the association between SCP and children’s (1) externalizing behavior, (2) internalizing behavior, and (3) school performance. These meta-analyses synthesized 21 studies (120 effect sizes; N = 67,400), 14 studies (18 effect sizes; N = 39,917), and 20 studies (47 effect sizes; N = 977,367), respectively. Studies were synthesized using a three-level approach to meta-analysis. The results revealed that SCP is positively associated with externalizing behavior (r = 0.27, *p* < 0.001) and internalizing behavior of children (r = 0.16, *p* < 0.001), and negatively with children’s school performance (r = −0.11, *p* < 0.001). This review concludes that SCP is a risk factor for externalizing behavior, internalizing behavior, and reduced school performance of children. Other techniques than SCP should be used for class management, and we recommend psychoeducational programs for schools and the wider community in which corporal punishment is still used. These programs should convey the detrimental effects of SCP and alternative discipline techniques. More awareness of the detrimental effects of SCP is needed to make the school environment a safe place for all children across the world.

## 1. Introduction

School corporal punishment (SCP) is widely used around the world for disciplining children [1]. Despite a shift towards the prohibition of corporal punishment in schools across many countries, it is still a lawful discipline strategy in 64 countries, including the Australian states of Queensland and Western Australia, and 19 states of the United States of America [2]. Furthermore, even in countries where SCP is not allowed, prevalence studies show that SCP still occurs [3]. Two reviews have already shown that SCP has detrimental effects on children’s development [1,3], but no statistical summary of the effects of SCP was yet available. Therefore, the primary goal of this study was to conduct a meta-analytic review of the associations between SCP and three child outcomes: internalizing problems, externalizing problems, and school performance. A second aim was to study how overall associations between SCP and these child outcomes are moderated by sample and study characteristics, such as sex of pupils, age of pupils, and the operationalization of SCP in primary studies.

Corporal punishment (CP) can be defined as “any punishment in which physical force is used and intended to cause some degree of pain or discomfort, however light” [4]. Examples are hitting, kicking, and shaking a child, but also pulling a child’s hair, and forcing children to stay in uncomfortable positions. SCP is a form of CP applied by teachers or other school staff, and not by other children. Teachers or other school staff may use their hands to punish children, but may also use objects such as a belt, a wooden stick, or a shoe. Qualitative studies show that children are corporally punished in school for a variety of behaviors, such as fighting with other children [5], coming late to class, disturbing the class, academic failure [6], not wearing the school uniform, failure of parents to pay school fees [7], not following school rules, not writing properly, and not having the right equipment [8]. All pupils in a class can also be subjected to corporal punishment at once for instance because they perform academically poorly, or because one pupil disrupts the classroom [9].

Several issues arise when SCP is inflicted upon a child. First, SCP conflicts with several children’s rights and in particular with article 19 of the Convention of the Rights of the Child [10] stating that States Parties should, among other things, protect children from all forms of physical violence. Furthermore, article 28 of that same convention states that States Parties should ensure that school discipline should be used in line with the child’s human dignity. When SCP is inflicted, children are not protected from physical violence, and SCP causes feelings of indignity [7]. Thus, SCP conflicts with multiple children’s rights. On top of this rights-based perspective, research shows that children tend to be repeatedly punished by a teacher [11], raising the question about SCP’s effectiveness. After all, one could argue that SCP is effective when the pupil stops his disruptive behavior and SCP does not have to be inflicted again. Relatedly, teachers using SCP do generally not explain to children why their behavior is undesired and how they should behave [9], which, in turn, makes it unlikely that children will positively change their behavior. Fourth, research shows that children will change their behavior rather out of fear for CP [12] than out of respect for the teacher and an understanding of the behavioral norms [13]. Fifth, meta-analyses of the outcomes of CP inflicted on children by their parents or caregivers in the home environment found detrimental effects on children’s behavior and cognitive functioning [14,15,16,17].

Several theories can explain why the use of SCP is associated with negative outcomes for children. First, from the perspective of social-learning theory [18], exposure to SCP can increase children’s externalizing behavior, which is defined as outwardly directed behavior (e.g., aggression, conduct problems, hyperactivity, hostile behavior [19]). By observing and imitating behaviors expressed by significant others, a child tends to learn that being aggressive is an acceptable response to others that do not behave in the way the child desires [18]. Thus, teachers or school staff inflicting corporal punishment on a child indirectly approve aggressive behavior. In turn, it is likely that the child internalizes aggression as an acceptable way to react, and therefore tends to show aggressive behaviors to others. A previous study from Nigeria indeed showed that school corporal punishment was a significant risk factor for physical aggressive behavior of boys [20]. Thus, instead of reducing children’s disruptive behavior by using SCP, it is far more likely that children’s disruptive behavior increases.

From an extended attachment theory perspective [21], it can be expected that exposure to SCP increases children’s internalizing behavior, which is defined as inwardly directed behavior in which emotions and feelings are overcontrolled and unregulated (e.g., anxiety, depression, social inhibition, and psychosomatic complaints; [19]). Although the teacher–student relationship differs from a parent–child relationship because it is not exclusive and more short-term [22], teachers can serve as an attachment figure for children. When student–teacher relationships are positive (i.e., relationships are characterized by a high level of closeness and a low level of conflict), children generally feel emotionally secure. As a consequence, they dare to explore the classroom environment [23]. On the other hand, negative student–teacher relationships (i.e., relationships characterized by high levels of conflict and low levels of closeness) are associated with less secure children [23] with lower levels of self-worth [24]. When children are corporally punished by their teachers, a negative student–teacher relationship may develop. This, in turn, decreases their emotional security and, therefore, increases their internalizing behavior problems (such as anxiety, depression, and social inhibition). A cross-sectional study on Indian secondary school children indeed showed that the more children are exposed to school corporal punishment, the more internalizing problems they have [25].

From the same theoretical perspective [21], it can also be expected that exposure to SCP reduces children’s school performance. A negative teacher–student relationship may result in a lower emotional security [23], which redirects energy from academic tasks [26]. Consequently, this hampers and interferes with natural efforts to be involved and engaged in academic tasks and, thus, school performance decreases (i.e., obtaining lower school grades [26]).

Further, from a biopsychosocial perspective, it can be expected that SCP negatively affects children’s cognitive development as a result of stress. Children exposed to CP may perceive this as stressful [27]. As stress negatively influences a child’s brain structure and development, it also affects a child’s overall functioning [28]. This is in line with previous research on parental corporal punishment showing that CP changes children’s brain structure [29]. Thus, children exposed to SCP may have impaired cognitive functioning due to stress-associated changes in brain development. A study on Jamaican primary school children showed that the more exposed children are to school corporal punishment, the lower they score on spelling skills, mathematics performance, and reading tests [30]. Thus, theory as well as empirical findings indicate negative effects of SCP.

The main reason for teachers applying SCP is the belief that SCP is needed to make reoccurrences of undesirable behavior less likely [1]. Further, because SCP was an acceptable teacher discipline strategy for multiple decades, teachers themselves may have internalized SCP as proper teacher behavior. This may be due to their own victimization of SCP or because they have witnessed SCP as a child, resulting in their acceptance of SCP as a proper discipline strategy [31] and having positive attitudes towards SCP [32]. Engaging in SCP or other inappropriate responses to children’s behavior may also be the consequence of teachers experiencing stress [32] that can be due to, for instance, underpayment, large classes, and poor school resources [7]. Furthermore, a teacher’s lack of knowledge of the adverse consequences of SCP on one hand and alternative more positive discipline methods on the other contributes to teachers engaging in SCP [31].

Until now, the evidence for adverse effects of SCP was scattered and not meta-analytically synthesized so that robust estimates of the true impact of SCP were not yet available. This study aimed to fill this gap by synthesizing results of primary studies on the association between SCP and three developmental outcomes for children: (1) externalizing behavior problems; (2) internalizing behavior problems; and (3) school performance. We focused on these particular outcomes because they have been addressed most by primary researchers examining the effects of SCP. Consequently, a sufficient number of studies on each of these outcomes was available for a quantitative synthesis, so that a separate meta-analysis could be conducted for each of these three outcomes. A second aim was to study how the overall association between SCP and each outcome are moderated by sample and study characteristics, such as gender of the child, age of the child, and the way in which SCP was operationalized in primary studies. As for the overall association between SCP and each outcome, we expected in line with previous reviews [1,3] that exposure to SCP (1) increases children’s externalizing behavior problems as can be derived from social-learning theory [18], (2) increases children’s internalizing problems as can be derived from the extended attachment theory [25], and (3) deteriorates children’s school performance due to stress as can be derived from the extended attachment theory and the biopsychosocial perspective on child development [25,27].

## 2. Materials and Methods

### 2.1. Inclusion and Exclusion Criteria

Primary studies meeting the following six criteria were included. First, studies had to be written in English. Second, studies had to be published in peer reviewed scientific journals or as a (non-)governmental research report. Third, studies had to compare children exposed to SCP with children not exposed to SCP. Fourth, studies had to examine at least one of the three outcomes of interest (i.e., externalizing behavior, internalizing behavior, and/or school performance). Fifth, studies had to report at least one correlation, or sufficient statistical information to calculate at least one correlation (e.g., means and standard deviations, or odds ratios). Sixth, studies had to be quantitative in nature with either a cross-sectional or longitudinal design. Both retrospective and prospective studies were included.

Studies were excluded if they (1) were qualitative in nature (e.g., interviews), (2) SCP was not studied at the individual child level, but at the school or state level on for instance SCP allowance [33,34,35], or (3) reported on a sample other than children (e.g., teachers, [36,37,38]). No restrictions were set to the country in which a primary study was conducted, so that the most extensive synthesis of worldwide literature was possible. 

### 2.2. Search Strategy

To find studies eligible for inclusion, a structured literature search was performed in the electronic databases PsycINFO, ERIC, Web of Science, and Google Scholar. The full search string with all keywords that were used in searching these databases can be found in Section S1 of the online supplemental material. The three outcomes of interest (externalizing behavior, internalizing behavior, and school performance) were not specifically entered as keywords in the databases, as it was expected that these outcomes were not explicitly described in titles or abstracts of eligible primary studies. Further, reference lists of two review studies on school corporal punishment [1,3] were checked for relevant references that did not come up in the electronic search. Third, key researchers (e.g., Rami Benbenishty, Tobias Hecker, and Elizabeth Gershoff) in the field of SCP were emailed to ask about unpublished but relevant studies. Finally, reference lists of all primary studies were screened to find additional studies that were eligible for inclusion. To decide whether identified studies in the search strategy met the inclusion and exclusion criteria, titles, abstracts, and, if necessary, full texts were read. Studies were included until May 2021. At the start of the search procedure, 1834 potentially relevant studies were identified by searching electronic databases and an additional 37 potentially relevant studies were identified through other sources. In the end, 29 studies met the inclusion and exclusion criteria and were therefore included in the current review (see Figure S1 of Section S2 for a detailed flow diagram of the search procedure). Characteristics of the included studies can be found in Table S1 of Section S3 and the references of these studies can be found in Section S4.

### 2.3. Data Coding

Based on previous studies about SCP and CP in the home environment, a coding scheme was created (see Section S5) to code effect sizes and potential moderating variables that may influence the overall associations. Data were directly coded into SPSS. Prior to coding all included studies, two studies were coded and discussed with all authors of this study and a Research Master’s student that was trained in coding studies to adjust and finalize the coding scheme until full consensus on the coding scheme was reached. Next, nine studies were randomly selected and coded independently by the first author and the trained Research Master’s student to determine the interrater agreement. Before discussion between the coders, the interrater agreement was 91.7% for the number of effect sizes that could be extracted from primary studies and 69.4% for effect size calculations. For the coded study characteristics, the agreement was 84.9% and for the sample characteristics, the agreement was 89.7%. As we aimed for a perfect interrater agreement, any inconsistency in study coding was discussed and resolved. Most coding inconsistencies were due to misreading an article text or typing errors. In case where the first author and the trained student could not reach consensus, the other authors were consulted to discuss the discrepancy and to reach a final decision to which all authors agreed.

As SCP and outcome variables were operationalized differently across included studies, an important aspect of the coding procedure was to code how SCP and outcome variables were operationalized. Based on definitions and information reported in all included studies, we created four categories for SCP operationalization that differed in how children were physically punished in primary studies: (1) with the hands and/or an object, (2) without any object, (3) only with one or more objects, or (4) unknown, when a study did not further define physical punishment. As for the operationalization of outcome variables, we also added three discrete variables to the coding scheme, so that we were able to code the operationalization of externalizing and internalizing behavior problems, and school performance. For externalizing behavior we created ten categories in which studies could be classified according to the definitions or information they reported (i.e., bullying/peer perpetration, being verbally aggressive, being physically aggressive, being physically and verbally aggressive, having conduct disorder or conduct problems, being hyperactive, being delinquent or showing rule-breaking behavior, being stubborn, often telling lies, and a total scale score on the Strengths and Difficulties Questionnaire). For the operationalization of internalizing problems we created five categories (i.e., being depressed, being anxious, being depressed and anxious, having emotional problems, and having low self-esteem), and seven categories were created for type of school performance as reported in primary studies (i.e., spelling skills, reading skills, vocabulary score, mathematics performance, number of children repeating a school year or retaking exam(s), grade average, and an aggregated index of language, reading, and mathematics skills).

A meta-analysis on corporal punishment by parents showed that CP impacts older children more than younger children [14]. Further, SCP turns out to be more prevalent in boys than in girls and inflicted upon boys more severely than upon girls [39]. Therefore, it can be expected that children’s age and their gender significantly moderate the associations between SCP and externalizing behavior, internalizing behavior, and/or school performance. Therefore, the mean, minimum, and maximum age of the sample was recorded for each study. Regarding gender, the percentage of boys in the sample was recorded. We also coded the type of school participants were attending (only primary, only secondary, or mixed sample). 

The severity of school corporal punishment was coded as a previous meta-analysis on the impact of corporal punishment in the home environment of children showed weaker effects for children who are spanked than for children who are exposed to more severe forms of corporal punishment [14]. After screening the studies that were eligible for inclusion, we created four categories for SCP severity: SCP with hand(s) and/or object(s), SCP without any object, SCP only with object(s), and SCP not further defined.

We aimed to synthesize unadjusted or bivariate effect sizes as much as possible, as primary researchers often use different covariates in their analyses across primary studies. However, it was expected that some primary studies only reported adjusted effect sizes. We decided to also include these studies so that a maximum of information could be included in the current review. Ferguson [14] showed that associations between CP and negative long-term outcomes are stronger for unadjusted effects than for adjusted effects. Therefore, each included effect size was coded as unadjusted or adjusted, so that statistical adjustment could be tested as a moderator on the association between SCP and one of the three outcomes of interest in this review.

Several other variables were coded as well and tested as moderators. These variables were: (1) the time period in which a primary study measured SCP occurrence and/or frequency (e.g., last week, last month, last year); (2) the dimension of SCP examined in a primary study (i.e., presence versus absence of SCP and SCP frequency whether or not combined with different forms of SCP); (3) study design in terms of cross-sectional versus longitudinal studies; (4) the type of instrument that was used for measuring SCP and the outcomes (i.e., questionnaire, interview, or observation); (5) whether or not a primary study was published or unpublished; and (6) the continent in which a study was performed. Finally, some variables were coded for descriptive purposes, such as the publication year of studies, and the age range of primary study samples.

### 2.4. Data Analysis

As the first aim was to study the association between school corporal punishment (SCP) and three separate outcomes (i.e., externalizing behavior, internalizing behavior, and school performance), an overall association was estimated for each of these outcomes in three separate meta-analyses. The second aim was to study whether and how these overall associations between SCP and one of the outcomes are influenced by sample characteristics (e.g., sex of pupils, age of pupils) or study characteristics (e.g., study design, and the SCP dimension that was measured). Therefore, most of the coded variables were tested as potential moderators in each of the three meta-analyses. In analyzing the data, we based our strategy on the procedures for data analyses outlined in previous reviews in which a multilevel approach to meta-analysis was employed, i.e., [40,41] as well as a tutorial for performing a three-level meta-analysis [41].

We considered the included primary studies as a random sample of the population of studies, and therefore we performed random-effects meta-analyses [42,43]. The random-effects model assumes that differences in effect sizes across primary studies can be ascribed to methodological differences, so that not only within-study sampling error is present (i.e., variation in effect sizes due to chance), but also between-study variance (i.e., variation in effect sizes due to methodological differences). 

As primary studies tend to report on multiple dimensions and/or operationalizations of SCP, multiple relevant effect sizes could often be extracted from individual studies. However, in doing so, dependency in the data is introduced as effect sizes extracted from the same studies are more alike than effect sizes extracted from different studies. To deal with this dependency, the effect sizes were analyzed in three-level random effects meta-analytic models. This multilevel approach to meta-analysis may be preferred above more traditional meta-analytic techniques, as a maximum of information from primary studies can be retained in the synthesis, implying that a more valid estimate of the overall association can be obtained and more statistical power can be achieved. Moreover, homogeneity of effect sizes within studies is not assumed [44,45,46,47]. In three-level meta-analytic models, three types of variance are considered to model effect size dependency: sampling variance of effect sizes distributed at level 1 (i.e., variance between participants on individual effect sizes in individual studies); variance between effect sizes extracted from the same study distributed at level 2 (i.e., within-study heterogeneity); and variance between studies distributed at level 3 (i.e., between-study heterogeneity). 

Prior to moderator analyses, two one-sided log-likelihood ratio tests were performed for testing heterogeneity of within-study and between-study variance. If log-likelihood-ratio tests indicate significant differences in effect sizes within or between studies, more variability in effect sizes is present than can be expected based on sampling variance alone. Besides performing log-likelihood ratio tests for examining heterogeneity, the 75% rule of Hunter and Schmidt was used [48] that considers effect size variance to be substantial if less than 75% of the total amount of variance is distributed at level 1 (i.e., sampling variance). We used this rule as log-likelihood-ratio tests tend to have less statistical power to detect substantial within-study and between-study variability when a dataset consists of a relatively small number of primary studies and/or effect sizes [49].

In case of substantial and/or significant variance at level 2 or 3 of the model, the coded variables were tested individually as moderators. We only tested coded variables as moderators if (categories of) a potential moderator was based on at least three studies. In this way we minimized the number of moderator analyses to control the type 1 error rate. In addition, this criterion was used to ensure a minimum of statistical power in the analyses, as reliable methods for determining power in moderator analyses in three-level meta-analytic models are not yet available [50]. Only for the operationalizations of the three outcomes of interest and the time period in which SCP was measured did we not collapse categories and allowed categories that were based on less than three studies, as the categories were quite distinct in nature. Moreover, for these specific potential moderating variables, we aimed to run the moderator analysis in which information was retained as much as possible. To examine the significance of potential moderating variables, omnibus tests were performed, as log-likelihood-ratio tests cannot be used for this purpose [45]. Each potential moderating variable was tested in a separate model. A significant omnibus test implied that the tested variable significantly moderated the association between SCP and one of the three outcomes of interest. Prior to testing moderator variables, dummy codes were created for categorical variables and continuous variables were centered at their mean.

The analyses were carried out in R [51] using the rma.mv function of the metafor package [52] and with the syntax as outlined by Assink and Wibbelink [49]. As correlations are restricted in their range and do not have a normal sampling distribution, they were first transformed to Fisher’s z scores. After the analyses, Fisher’s z scores were converted back to correlations for ease of interpretation. We used α = 0.05 as the criterion for significance. Test statistics and CIs were based on the t-distribution [53] to reduce the inflation of type 1 errors. CIs were provided for regression coefficients, to give an indication of the precision of the estimated effect [54]. To estimate parameters, Restricted Maximum Likelihood estimation method (REML) was used. For the three outcomes, all tested moderators as well as significant and non-significant results of all moderator analyses are presented in Table S1 of Section S6.

To examine whether (a form of) bias was present in the estimated overall association between corporal punishment and externalizing problems, internalizing problems, and school performance, respectively, two bias assessment analyses were conducted. First, the funnel-plot-based trim and fill method was conducted [55,56], in which the symmetry of the effect size distribution was tested and restored in case of an asymmetrical distribution of effect sizes (i.e., an asymmetrical funnel plot), by imputing effect size estimates from “missing” studies. Second an adapted Egger’s test was conducted in which effect sizes were regressed on their standard errors in a 3-level meta-analytic model. In this adapted test, effect size dependency was accounted for, and a significant slope was an indication of bias. Both bias assessment analyses were performed in the R environment [51] with the functions “trimfill” and “rma.mv” of the metafor package [52].

## 3. Results

### 3.1. Externalizing Behavior Problems

The meta-analysis on externalizing behavior problems comprised 21 independent studies that reported on 120 effect sizes, and analyzed N = 67,400 participants. Studies were conducted in Asia (11), Africa (6), South America (2), Europe (1), and Africa/Asia (i.e., Egypt, 1). The mean age of the sample was 13.52 years (SD = 1.61). Studies operationalized SCP in terms of physical punishment using hands and/or an object (12 studies and 35 effect sizes; 57.1% and 29.2%, respectively), physical punishment without object(s) (6 studies and 72 effect sizes; 28.6% and 60%, respectively), and physical punishment only with object(s) (2 studies and 7 effect sizes; 9.5% and 5.8%, respectively). Two studies (9.5%) producing six effect sizes (5%) did not further define the school corporal punishment. In their studies, the primary researchers used the labels physical victimization (six studies; 28.6%), corporal punishment, physical punishment, or physical maltreatment (nine studies; 42.9%), physical violence (four studies; 19%), physical discipline (one study; 4.8%), or aggressive behavior (one study; 4.8%) to refer to SCP. A significant and positive overall association was found between school corporal punishment and externalizing behavior problems, r = 0.27, 95% CI (0.23; 0.31), t(119) = 12.17, *p* < 0.001, indicating that more exposure to school corporal punishment is associated with more externalizing behavior problems. Egger’s test was not significant, β = −1.41, t(118) = −1.11, *p* = 0.272, providing no indication for funnel plot asymmetry. However, the trim-and-fill procedure revealed that 21 effect sizes were missing on the left side of the funnel plot (see Figure S1 of Section S7). This indicates that the estimated mean association may be an overestimation of the true effect. The adjusted correlation after the “missing” effect sizes were imputed indeed showed a smaller, but still significant, correlation between SCP and externalizing problem behavior, r = 0.23, 95% CI (0.18; 0.28), t(140) = 9.00, *p* < 0.001. 

The three-level model had a significantly better fit than a two-level model without within-study variance, Δχ^2^(1) = 756.52, *p* < 0.001, and a two-level model without between-study variance, Δχ^2^(1) = 60.31, *p* < 0.001. Additionally, the estimated within-study variance and between-study variance components were greater than zero, τ^2^ = 0.004 and τ^2^ = 0.009, respectively. Further, 29.41% of the total variation was distributed to within-study variance and 68.43% of the total variation was distributed to between-study variance. Thus, because of substantial and significant variance at levels 2 and 3 of the model, a three-level model should be fitted to the data. Moderator analyses were conducted in an attempt to explain this within- and/or between-study variance (see Table 1 for significant results and Table S1 in Section S6 for all significant and non-significant results).

#### Moderator Analyses for SCP and Externalizing Behavior Problems

No significant moderating effects were found for any of the tested sample characteristics. As for study characteristics, two moderator variables were identified. First, the dimension of SCP had a significant moderating effect on the association between SCP and externalizing behavior problems. Studies that measured the frequency of SCP produced stronger associations, r = 0.31 (*p* < 0.001), than studies that measured the occurrence (i.e., presence versus absence) of SCP, r = 0.19 (*p* < 0.001). Second, the overall association was lower when externalizing behavior problems were measured in terms of delinquency and rule-breaking behavior, r = 0.26 (*p* < 0.001), than when bullying behavior was measured, r = 0.36 (*p* < 0.001). 

### 3.2. Internalizing Behavior Problems

The meta-analysis on the association between school corporal punishment and internalizing behavior problems comprised 14 independent samples, reporting on 18 effect sizes, and a total sample of N = 39,917 individuals. Studies were conducted in Asia (9), Europe (2), Africa (1), North America (1), and South America (1). The mean age of the sample was 13.98 years (SD = 2.41). Studies operationalized SCP in terms of physical punishment using hands and/or an object (9 studies and 10 effect sizes; 64.3% and 55.6%, respectively) and physical punishment only with object(s) (1 study and 2 effect sizes; 12.5% and 11.1%, respectively). Four studies (28.6%) producing six effect sizes (33.3%) did not further define the school corporal punishment. In their studies, the primary researchers used the labels (school) corporal punishment (six studies; 42.9%), physical punishment or maltreatment (four studies; 28.6%), (teacher) physical violence (two studies; 14.3%), physical punishment or conflict with teachers (one study; 7.14%) or aggressive behavior of teachers (one study; 7.14%) to refer to SCP. The three-level meta-analytic intercept-only model showed a significant positive association between school corporal punishment and internalizing behavior problems, r = 0.16, 95% CI (0.12; 0.19), t(17) = 8.61, *p* < 0.001, indicating that more exposure to school corporal punishment is associated with more internalizing behavior problems. Egger’s test was not significant, β = 2.37, t(16) = 2.11, *p* = 0.051, indicating the funnel plot was symmetric. This is in line with the trim-and-fill results showing that zero studies were missing on the left side of the funnel plot (see Section S7, Figure S2). Thus, both bias assessment methods did not provide indications for publication bias.

The three-level model had a significantly better fit than a two-level model without within-study variance, Δχ^2^(1) = 17.14, *p* < 0.001, but did not significantly fit better than a two-level model without between-study variance, Δχ^2^(1) = 0.00, *p* = 0.500. The estimated within-study variance was greater than zero, τ^2^ = 0.005. Further, 93.68% of the total variation was distributed to within-study variance and <0.01% of the total variation was distributed to between-study variance. Thus, there was significant and substantial (according to the 75% rule of Hunter and Schmidt [48]) within-study variance. Therefore, a three-level model should be fitted to the data and moderator analyses were conducted in an attempt to explain this within-study variance (see Table 1 and Table S1 of Section S6).

#### Moderator Analyses for SCP and Internalizing Behavior Problems

As for sample characteristics, one significant moderator effect was found. Mean age of the sample had a moderating effect on the association between SCP and internalizing behavior problems. As the mean age of the sample increased, the overall strength between SCP and internalizing behavior problems decreased, β = −0.02 (*p* = 0.037). 

None of the tested study characteristics was a significant moderator. We decided not to test the type of instrument for measuring SCP nor the type of instrument for measuring internalizing behavior problems as moderator, as interviews were generally conducted with younger children to assess SCP and internalizing behavior problems. A moderating effect of type of instrument would likely be caused by the age of the child rather than the type instrument.

### 3.3. School Performance

The meta-analysis on the association between school corporal punishment and school performance comprised 20 independent samples, reporting on 47 effect sizes, and a total sample of N = 977,367 individuals. Studies were conducted in Asia (six), Africa (five), North America (four), South America (three), Europe (one), and Africa/Asia (i.e., Egypt, one). The mean age of the sample was 11.99 years (SD = 3.27). Studies operationalized SCP in terms of physical punishment using hands and/or an object (16 studies and 37 effect sizes; 80% and 78.7%, respectively) and physical punishment without objects (1 study and 3 effect sizes; 5% and 6.4%, respectively). Three studies (15%) producing seven effect sizes (14.9%) did not further define the school corporal punishment. In their studies, the primary researchers used the labels corporal punishment (seven studies; 33.3%), physical punishment (nine studies; 45%), (teacher) physical violence (three studies; 15%), or teacher–student violence (one study; 5%) to refer to SCP. A three-level meta-analytic intercept-only model showed a significant negative association between school corporal punishment and school performance, r = −0.11, 95% CI (−0.15; −0.07), t(46) = −5.31, *p* < 0.001, indicating that more exposure to school corporal punishment is associated with reduced school performance of children. Egger’s test was significant, β = −3.57, t(45) = −3.38, *p* = 0.002, indicating asymmetry in the funnel plot of effect sizes. This asymmetry was also found by the trim-and-fill algorithm, which revealed that four effect sizes were missing on the right side of the funnel plot (see Section S7, Figure S3). This indicates that the estimated (negative) mean effect may be an overestimation of the true effect. The adjusted correlation after the “missing” effect sizes were imputed indeed showed a smaller, but still significant, negative correlation between SCP and school performance, r = −0.09, 95% CI (−0.14; −0.04), t(50) = −3.91, *p* < 0.001. Thus, these results indicate that bias may have affected the results of this meta-analysis.

The three-level model had a significantly better fit than a two-level model without within-study variance, Δχ^2^(1) = 29.30, *p* < 0.001 and a two-level model without between-study variance, Δχ^2^(1) = 57.20, *p* < 0.001. In addition, the between-study variance was greater than zero, τ^2^ = 0.008. Further, 0.21% of the total variation was distributed to within-study variance and 99.56% of the total variation was distributed to between-study variance. Thus, moderator analyses were conducted in an attempt to explain the within- and between-study variance (see Table 1 and Table S1 of Section S6).

#### Moderator Analyses for SCP and School Performance

No significant moderators were found for any of the tested study or sample characteristics.

## 4. Discussion

In this meta-analytic review, a positive and significant association was found between SCP and externalizing behavior problems of children (r = 0.27), and between SCP and internalizing behavior problems of children (r = 0.16). A negative and significant association was found between SCP and children’s school performance (r = −0.11). Taken together, these results indicate that exposure to SCP is a risk factor for externalizing behavior problems, internalizing behavior problems, and reduced school performance of children.

SCP was stronger related to externalizing behavior problems than to internalizing behavior problems and school performance, which is in line with findings of a recent meta-analysis on the effects of corporal punishment in the home environment of children [16]. This may be explained by the overlap in children’s externalizing behavior and the behavior of teachers inflicting corporal punishment upon children (i.e., physical aggression). By being exposed to SCP and observing SCP, children may learn that SCP is an appropriate discipline method and an acceptable way to react (cf. social learning theory of Bandura [18]). In turn, children may mimic the teacher’s behavior and as a result show more aggressive behavior toward others. The results showed that SCP is also a risk factor for internalizing problems and reduced school performance, as can be derived from the extended attachment theory [25] and a biopsychosocial perspective on child development [27]. Both outcomes are, however, less reflected in the physical act of school corporal punishment itself. 

The bias assessment methods showed some indication for publication bias in the meta-analysis on externalizing behavior problems implying that the estimated mean association may be an overestimation of the true effect. In contrast, there were indications for selection bias in the meta-analysis on school performance, as the analyses revealed that the estimated mean association may be an overestimation of the true effect. Both associations should thus be interpreted with some caution. Nevertheless, after imputing the “missing” effect sizes and re-estimating the mean associations, both “adjusted” associations were still significant and did not differ much from the initially estimated mean associations. Furthermore, the bias assessment methods that we applied have their own limitations: The performance of the trim-and-fill method is limited when the effect size distribution is heterogeneous [57], and the statistical power of the Egger’s test decreases as the number of included studies and effect sizes decrease [58].

Several moderating variables were identified. First, for internalizing behavior problems, we found that the strength of the association with SCP decreases as the mean age of the children in samples increases. This finding may be explained by underdeveloped emotion regulation skills in younger children [59]. Younger children with emotion regulation skills that have not yet been fully mastered are more prone to developing internalizing behavior problems. In older children exposed to SCP, the association between SCP and internalizing problems may be smaller as their emotion regulation process was not hampered, or to a lesser extent. As a result, the impact of SCP on internalizing behavior problems may be larger in younger children than in older children. It should be noted, however, that the included studies did not report on the age at which children were first exposed to corporal punishment by school staff. Future researchers examining the impact of SCP on children may want to take this variable into account.

Second, no moderating effect of gender was found in any of the three meta-analyses, implying that the impact of SCP on children’s internalizing problems, externalizing problems, and school performance does not seem to differ between boys and girls. Although a previous review [3] led us to expect that the impact of SCP would be stronger for boys, the results did not confirm this. However, the results are in line with meta-analyses on CP in the home environment that neither found differences in impact of CP between boys and girls [15,17].

Several study characteristics that were tested as moderators should be discussed. First, type of punishment was not identified as a moderator, which is not in line with a previous meta-analysis on CP in the home environment that found stronger effect sizes for CP compared to spanking [14]. However, in the primary studies that were synthesized in the current review, no effect sizes for specific punishments such as spanking were (separately) reported. Therefore, the type of punishment was coded in terms of how SCP was operationalized: using a hand and/or object, without any object, only with objects, or as not further defined. It might be argued that not using any object reflects spanking, but this might also refer to other forms of corporal punishment such as kicking, hitting, and slapping, as all these forms do not involve objects. Thus, although the current results do not provide insight into the impact of spanking, it is quite plausible that this type of punishment in school is also associated with detrimental developmental outcomes, as is shown by a previous meta-analysis on CP in the home environment of children [14].

Second, the operationalization of externalizing behavior had a significant moderating effect in the sense that stronger effect sizes were found for peer perpetration and bullying than for delinquency and rule breaking behavior. It may be that both bullying and SCP occur in the classroom and that bullying may present itself as a form of physical aggression. On the other hand, delinquent and rule breaking behavior may be more prevalent outside the school setting and does not necessarily reflect physical aggression. When children are exposed to SCP or observe SCP inflicted on other children, they might start inflicting this aggressive behavior themselves upon other children (cf. social learning theory of Bandura [18]), whereas delinquent and rule breaking behavior might be a more indirect effect of SCP for which other risk factors are more important. 

Third, the association between SCP and externalizing problems was stronger when SCP was measured in terms of frequency than in terms of presence versus absence. Apparently, it is important for primary researchers to consider what SCP dimensions are to be measured, as this will affect the results. We recommend future researchers to take multiple dimensions of SCP into account, as asking children about the presence or absence of SCP may be too imprecise, but asking children about the frequency of SCP may particularly be prone to recalling errors.

Several limitations need to be addressed. First, no inferences about causality can be drawn, indicating that the current results do not make clear whether school corporal punishment leads to problematic child developmental outcomes or whether the reverse is true. The latter may be the case, as children can be physically punished for fighting with other children [5], disturbing the class (because of their externalizing behavior), and showing poor academic behavior [6]. Further, interviews with teachers show that they are more likely to use SCP when they experience stress [33], which could be caused by children disturbing the classroom. However, it is also likely that the associations are bidirectional. For example, when a child disrupts the classroom, the teacher could eventually inflict SCP upon the child, which will increase the child’s disruptive behavior in the future, and elicit the use of SCP, and so on. Previous research indeed found evidence for reciprocal relations between children’s aggressive behavior and teacher–child conflicts [60]. Children showing internalizing problems, on the other hand, generally develop a closer relationship with their teacher(s) as teachers might be more willing to help these children [61]. Therefore, it is more likely that SCP increases internalizing problems, rather than internalizing problems increase teachers’ use of SCP. 

Further, there were indications for bias in the meta-analyses on externalizing problems and school performance, meaning the results may have been inflated and deflated respectively, though to a small extent. Moreover, multiple coded variables could not be examined as a moderator, as primary studies were limited in their descriptions on (categories of) these variables. Finally, a multiple moderator model was not examined, because of the relatively small amount of data in all three meta-analyses, implying the statistical power would be low in examining such a model. Future primary researchers are therefore encouraged to report more elaborately on their results and the variables that they examined.

Despite these limitations, this was the first study to statistically summarize the impact of SCP on three developmental outcomes of children. As such, this review gives a more objective insight into the associations between SCP and child developmental outcomes than previously conducted reviews [1,3]. In addition, more insight was gained by examining variables that may affect the association between SCP and developmental outcomes, such as gender and age of children, and the way in which SCP was measured in primary studies. Further, by applying a three-level-approach to meta-analysis all relevant effect sizes in primary studies could be preserved and synthesized, as the effect size dependency is modeled in this approach. As a result, we could obtain more valid estimates of the overall associations than when a more traditional meta-analysis technique would have been used [45,46,47,48].

This meta-analysis implies that SCP is a risk factor for three adverse developmental outcomes in children, and as such, this review contributes to the worldwide discussion about whether SCP should be allowed or prohibited. Our results favor the opponents of SCP. The findings can be used to inform and advice policymakers, child welfare professionals, and teachers. Previous research suggests that teachers’ lack of knowledge of the potential consequences of SCP, on the one hand, and not knowing about alternative and more positive discipline methods on the other, contributes to using SCP as a “positive” discipline method [31]. We therefore suggest that the current findings are incorporated into psychoeducational programs that train teachers in effective and ineffective discipline methods for children. As the use of CP is usually embedded in the wider community than just school teachers, these programs should target the broader social environment of children, including parents and caretakers. In future research it is necessary to investigate the effectiveness of these programs in order to reduce SCP and enhance the child’s development.

## 5. Conclusions

School corporal punishment (SCP) is still a widely used and legal practice in many countries for disciplining children. This review shows that the infliction of SCP upon children is associated with externalizing behavior problems, internalizing behavior problems, and reduced school performance. These findings stress that other techniques than SCP should be used for class management. Awareness of its detrimental effects is needed to make the school environment a safe place for all children across the world.

## Figures and Tables

**Table 1:** Significant Results for Categorical and Continuous Variables Tested as Moderators (in Bivariate Models).

Variables Tested as Moderators	# S	# ES	Intercept (95% CI)/ Mean *Z* (95% CI)	Mean *r*	β (95% CI)	*F* ^a^	*p* ^b^
Externalizing behavior problems							
Study characteristics							
SCP dimension that was measured						5.058	0.008 **
Frequency (RC)	8	80	0.314 (0.258; 0.369) ***	0.304			
Present versus absent	7	22	0.191 (0.126; 0.256) ***	0.189	−0.123 (−0.208; −0.037) **		
Frequency and types	6	18	0.321 (0.251; 0.390) ***	0.310	0.007 (−0.082; 0.096)		
Operationalization externalizing behavior						2.600	0.009 **
Delinquency/rule-breaking behavior (RC)	11	36	0.267 (0.217; 0.318) ***	0.261			
Physical aggression	11	31	0.293 (0.241; 0.345) ***	0.285	0.026 (−0.006; 0.058)		
Verbal aggression	8	28	0.261 (0.208; 0.315) ***	0.256	−0.006 (−0.039; 0.027)		
Peer perpetration/bullying	7	7	0.375 (0.303; 0.447) ***	0.358	0.108 (0.028; 0.187) **		
Being physical and verbally aggressive	1	6	0.300 (0.136; 0.465) ***	0.291	0.033 (−0.139; 0.205)		
Total scale score on SDQ	2	4	0.178 (0.050; 0.305) **	0.176	−0.089 (−0.226; 0.048)		
Conduct disorder/conduct problems	3	3	0.192 (0.083; 0.300) ***	0.189	−0.075 (−0.184; 0.033)		
Hyperactivity	3	3	0.167 (0.058; 0.275) **	0.165	−0.101 (−0.209; 0.007)		
(Often) Telling lies	1	1	0.236 (0.098; 0.374) **	0.232	−0.031 (−0.161; 0.100)		
Being stubborn	1	1	0.149 (0.011; 0.287) *	0.148	−0.118 (−0.248; 0.013)		
Internalizing behavior problems							
Sample characteristics							
Mean age of the sample in years	10	12	0.179 (0.129; 0.229) ***	-	−0.021 (−0.041; −0.002) *	5.773	0.037 *

Note. # S = number of independent study samples; # ES = number of effect sizes; Mean *Z* = Mean effect size (Fisher’s *Z*); CI = confidence interval; Mean *r* = Mean effect size (Pearson’s correlation); β = estimated regression coefficient. ^a^ Omnibus test of all regression coefficients in the model. ^b^ *p*-value of the omnibus test. SCP = School corporal punishment; SDQ = Strengths and Difficulties Questionnaire; RC = Reference category. Non-significant results can be found in Section S6. * *p* < 0.05; ** *p* < 0.01; *** *p* < 0.001.

## Data Availability

The dataset analyzed in this review is available from the corresponding author (M.Assink@UvA.nl) on reasonable request.

## Supplementary Materials

The following supporting information can be downloaded at: https://www.mdpi.com/article/10.3390/children9030383/s1, Section S1: Search strategies, Section S2: PRISMA flow diagram, Section S3: Characteristics of included studies, Section S4: References of included studies, Section S5: Coding scheme, Section S6: Significant and non-significant results of moderator analyses, Section S7: Funnel plots.

## References

[1] Gershoff E.T. (2017). School corporal punishment in global perspective: Prevalence, outcomes, and efforts at intervention. Psychol. Health Med..

[2] Global Partnership to End Violence Against Children (2021). Prohibiting All Corporal Punishment of Children: Laying the Foundations for Non-Violent Childhoods. https://endcorporalpunishment.org/wp-content/uploads/2021/04/Prohibiting-all-corporal-punishment-of-children-laying-the-foundations-for-nonviolent-childhoods.pdf.

[3] Heekes S.-L., Kruger C.B., Lester S.N., Ward C.L. (2020). A systematic review of corporal punishment in schools: Global prevalence and correlates. Trauma Violence Abus..

[4] UN Committee on the Rights of the Child (2006). General Comment No. 8: The Right of the Child to Protection from Corporal Punishment and Other Cruel or Degrading Forms of Punishment (arts. 19; 28, para. 2; and 37, inter alia). http://endcorporalpunishment.org/wp-content/uploads/key-docs/CRC-general-comment-8.pdf.

[5] Medway F.J., Smircic J.M. (1992). Willingness to use corporal punishment among school administrators in South Carolina. Psychol. Rep..

[6] Breen A., Daniels K., Tomlinson M. (2015). Children’s experiences of corporal punishment: A qualitative study in an urban township of South Africa. Child Abus. Negl..

[7] Elbla A.I.F. (2012). Is punishment (corporal or verbal) an effective means of discipline in schools? Case study of two basic schools in Greater Khartoum/Sudan. Procedia Soc..

[8] Morrow V., Singh R., Parkes J. (2015). Children’s perceptions of punishment in schools in Andhra Pradesh, India. Gender Violence in Poverty Contexts: The Educational Challenge.

[9] Pinheiro P.S. (2006). World Report on Violence against Children. United Nations. https://violenceagainstchildren.un.org/sites/violenceagainstchildren.un.org/files/document_files/world_report_on_violence_against_children.pdf.

[10] United Nations (UN) (1989). Convention on the Rights of the Child. https://www.ohchr.org/Documents/ProfessionalInterest/crc.pdf.

[11] Little S.G., Akin-Little A. (2008). Psychology’s contributions to classroom management. Psychol. Sch..

[12] Awoniyi F.C. (2021). Are caning and learning friends or foes in Ghanaian secondary schools?. Cogent. Educ..

[13] Brown B. (2009). Perceptions of student misconduct, perceived respect for teachers, and support for corporal punishment among school teachers in South Korea: An exploratory case study. Educ. Res. Policy Pract..

[14] Ferguson C.J. (2013). Spanking, corporal punishment and negative long-term outcomes: A meta-analytic review of longitudinal studies. Clin. Psychol. Rev..

[15] Gershoff E.T. (2002). Corporal punishment by parents and associated child behaviors and experiences: A meta-analytic and theoretical review. Psychol. Bull.

[16] Gershoff E.T., Grogan-Kaylor A. (2016). Spanking and child outcomes: Old controversies and new meta-analyses. J. Fam. Psychol..

[17] Paolucci E.O., Violato C. (2004). A meta-analysis of the published research on the affective, cognitive, and behavioral effects of corporal punishment. J. Psychol..

[18] Bandura A. (1973). Aggression: A Social Learning Analysis.

[19] Eisenberg N., Cumberland A., Spinrad T.L., Fabes R.A., Shepard S.A., Reiser M., Murphy B.C., Losoya S.H., Guthrie I.K. (2001). The relations of regulation and emotionality to children’s externalizing and internalizing problem behavior. Child Dev..

[20] Ani C., Grantham-McGregor S. (1998). Family and personal characteristics of aggressive Nigerian boys: Differences from and similarities with Western findings. J. Adolesc. Health.

[21] Ainsworth M.D., Blehar M.C., Waters E., Wall S. (1978). Patterns of Attachment: A Psychological Study of the Strange Situation.

[22] Verschueren K., Koomen H.M.Y. (2012). Teacher-child relationships from an attachment perspective. Attach. Hum. Dev..

[23] Thijs J.T., Koomen H.M.Y. (2008). Task-related interactions between kindergarten children and their teachers: The role of emotional security. Infant. Child Dev..

[24] Doumen S., Buyse E., Colpin H., Verschueren K. (2011). Teacher-child conflict and aggressive behaviour in first grade: The intervening role of children’s self-esteem. Infant. Child Dev..

[25] Deb S., Kumar A., Holden G.W., Simpson Rowe L. (2017). School corporal punishment, family tension, and students’ internalizing problems: Evidence from India. Sch. Psychol. Int..

[26] Boekaerts M. (1993). Being concerned with well-being and with learning. Educ. Psychol..

[27] Turner H.A., Finkelhor D. (1996). Corporal punishment as a stressor among youth. J. Marriage Fam..

[28] Shonkoff J.P., Garner A.S. (2012). The lifelong effects of early childhood adversity and toxic stress. Pediatrics.

[29] Tomoda A., Suzuki H., Rabi K., Sheu Y.-S., Polcari A., Teicher M.H. (2009). Reduced prefrontal cortical gray matter volume in young adults exposed to harsh corporal punishment. NeuroImage.

[30] Baker-Henningham H., Meeks-Gardner J., Chang S., Walker S. (2009). Experiences of violence and deficits in academic achievement among urban primary school children in Jamaica. Child Abus. Negl..

[31] Global Initiative to End All Corporal Punishment of Children (2017). Prohibiting Corporal Punishment in Schools: Answers to Frequently Asked Questions. https://endcorporalpunishment.org/wp-content/uploads/faqs/FAQ-EN-schools-updated-2019.pdf.

[32] Ssenyonga J., Hermenau K., Nkuba M., Hecker T. (2019). Stress and positive attitudes towards violent discipline are associated with school violence by Ugandan teachers. Child Abus. Negl..

[33] Arcus D. (2002). School shooting fatalities and school corporal punishment: A look at the states. Aggress. Behav..

[34] Han S. (2014). Corporal punishment and student outcomes in rural schools. Educ. Res. Policy Pract..

[35] Talwar V., Carlson S.M., Lee K. (2011). Effects of a punitive environment on children’s functioning: A natural experiment. Soc. Dev..

[36] Ahmad I., Said H., Awang Z., Yasin M., Hassan Z., Mansur S. (2014). Effect of self-efficacy on the relationship between corporal punishment and school dropout. Rev. Eur. Stud..

[37] Ahmad I., Said H., Khan F. (2013). Effect of corporal punishment on students’ motivation and classroom learning. Rev. Eur. Stud..

[38] Khoury-Kassabri M. (2012). The relationship between teacher self-efficacy and violence toward students as mediated by teacher’s attitude. Soc. Work Res..

[39] Youssef R.M., Attia M.S.E.D., Kamel M.I. (1998). Children experiencing violence II: Prevalence and determinants of corporal punishment in schools. Child Abus. Negl..

[40] Assink M., van der Put C.E., Meeuwsen M.W.C.M., de Jong N.M., Oort F.J., Stams G.J.J.M., Hoeve M. (2019). Risk factors for child sexual abuse victimization: A meta-analytic review. Psychol. Bull.

[41] Assink M., Van der Put C.E., Hoeve M., De Vries S.L.A., Stams G.J.J.M., Oort F.J. (2015). Risk factors for persistent delinquent behavior: A meta-analytic review. Clin. Psychol. Rev..

[42] Raudenbush S.W., Valentine L.V.H., Cooper J.C. (2009). Analyzing Effect Sizes: Random-Effects Models. The Handbook of Research Synthesis and Meta-Analysis.

[43] Van Den Noortgate W., Onghena P. (2003). Multilevel meta-analysis: A comparison with traditional meta-analytical procedures. Educ. Psychol. Meas..

[44] Cheung M.W.-L. (2014). Modeling dependent effect sizes with three-level meta-analyses: A structural equation modeling approach. Psychol. Methods.

[45] Hox J.J. (2010). Multilevel Analysis: Techniques and Applications.

[46] Van den Noortgate W., López-López J.A., Marín-Martínez F., Sánchez-Meca J. (2013). Three-level meta-analysis of dependent effect sizes. Behav. Res. Methods.

[47] Van den Noortgate W., López-López J.A., Marín-Martínez F., Sánchez-Meca J. (2015). Meta-analysis of multiple outcomes: A multilevel approach. Behav. Res. Methods.

[48] Hunter J.E., Schmidt F.L. (1990). Methods of Meta-Analysis: Correcting Error and Bias in Research Findings.

[49] Assink M., Wibbelink C.J.M. (2016). Fitting three-level meta-analytic models in R: A step-by-step tutorial. Quant. Meth. Psych..

[50] Griffin J.W. (2021). Calculating statistical power for meta-analysis using metapower. Quant. Meth. Psych..

[51] R Core Team (2020). R: A Language and Environment for Statistical Computing.

[52] Viechtbauer W. (2010). Conducting meta-analyses in R with the metafor package. J. Stat. Softw..

[53] Knapp G., Hartung J. (2003). Improved tests for a random effects meta-regression with a single covariate. Stat. Med..

[54] Cumming G. (2013). Understanding the New Statistics: Effect Sizes, Confidence Intervals, and Meta-Analysis.

[55] Duval S., Tweedie R. (2000). A nonparametric ‘trim and fill’ method of accounting for publication bias in meta-analysis. J. Am. Stat. Assoc..

[56] Duval S., Tweedie R. (2000). Trim and fill: A simple funnel-plot-based method of testing and adjusting for publication bias in meta-analysis. Biometrics.

[57] Nik Idris N.R. (2012). A comparison of methods to detect publication bias for meta-analysis of continuous data. J. Appl. Sci..

[58] Peters J.L., Sutton A.J., Jones D.R., Abrams K.R., Rushton L. (2007). Performance of the trim and fill method in the presence of publication bias and between-study heterogeneity. Stat. Med..

[59] Izard C.E., Youngstrom E.A., Fine S.E., Mostov A.J., Trentacosta C.J., Cicchetti D., Cohen D.J. (2006). Emotions and developmental psychopathology. Developmental Psychopathology: Theory and Method.

[60] Doumen S., Verschueren K., Buyse E., Germeijs V., Luyckx K., Soenens B. (2008). Reciprocal relations between teacher-child conflict and aggressive behavior in kindergarten: A three-wave longitudinal study. J. Clin. Child Adolesc. Psychol..

[61] Roorda D.L., Verschueren K., Vancraeyveldt C., Van Craeyevelt S., Colpin H. (2014). Teacher-child relationships and behavioral adjustment: Transactional links for preschool boys at risk. J. Sch. Psychol..

