# Supplementary material for: The Association between School Corporal Punishment and Child Developmental Outcomes: A Meta-Analytic Review
Source: Children (Basel). 2022 Mar 9;9(3):383. doi: 10.3390/children9030383 (PMC8946887; doi:10.3390/children9030383)
Supplement: Supplementary file 1 [file children-09-00383-s001.zip › children-1581899-supplementary.pdf]

Section S1

Search Strategies for Databases ERIC, PsycINFO, Web of Science, and Google Scholar

Search ERIC (05-03-2021)

#1 School setting

(School* or teacher* or ((staff or personnel) and (school or educat*)) or educator*).ti,ab,id. or teacher*.sh.

#2 Age group research population

children/ or preadolescents/ or youth/ or adolescents/ or early adolescents/ or late adolescents/ or elementary school students/ or middle school students/ or junior high school students/ or secondary school students/ or high school students/ or students/ or (Student* or pupil* or Child* or adolescent* or teen* or youth* or youngster*).ti,ab,id.

#3 Physical Punishment

(student victim* or corporal* punish* or physical* punish* or physical maltreat* or ((teacher* or staff or educator*) adj3 (violen* or maltreat* or punish*))).ti,ab,id. or punishment/

#4 Study type

Longitudinal studies/ or followup studies/ or (survey* or cross-sectional or longitud* or followup or follow-up or cohort* or questionnair* or task* or rating* or scale* or covariat* or correlat* or mediat* or moderat* or effect size or outcomes or odds).ti,ab,id.

1 AND 2 AND 3 AND 4 494 results

Search PsycINFO (05-03-2021)

#1 School setting

(School* or teacher* or ((staff or personnel) and (school or educat*)) or educator*).ti,ab,id. or educational personnel/

#2 Age group research population

school age 6 12 yrs or adolescence 13 17 yrs).ag. or (Student* or pupil* or Child* or adolescent* or teen* or youth* or youngster*).ti,ab,id.

#3 Physical Punishment

(student victim* or corporal* punish* or physical* punish* or physical maltreat* or ((teacher* or staff or educator*) adj3 (violen* or maltreat* or punish*))).ti,ab,id. or punishment/ or physical discipline/

#4 Study type

Longitudinal studies/ or Followup studies/ or prospective studies/ or retrospective studies/ or (followup study or longitudinal study or prospective study or retrospective study).md. or (survey* or cross-sectional or longitud* or followup or follow-up or cohort* or questionnair* or task* or rating* or scale* or covariat* or correlat* or mediat* or moderat* or effect size or outcomes or odds).ti,ab,id.

1 AND 2 AND 3 AND 4 745 results

Search Web of Science (05-03-2021)

#1 School setting

TS=("School*" OR "teacher*" OR (("staff" OR "personnel") AND ("school" OR "educat*")) OR "educator*")

#2 Age group research population

TS=(“Student*” OR “pupil*” OR “Child*” OR “adolescent*” OR “teen*” OR “youth*” OR “youngster*”)

#3 Physical Punishment

TS=(“student victim*” OR “corporal* punish*” OR “physical* punish*” OR “physical maltreat*” OR ((“teacher*” OR “staff” OR “educator*”) NEAR/2 (“violen*” OR “maltreat*” OR “punish*”)))

#4 Study type

TS=(“survey*” OR “cross-sectional” OR “longitud*” OR “followup” OR “follow-up” OR “cohort*” OR “questionnair*” OR “task*” OR “rating*” OR “scale*” OR “covariat*” OR “correlat*” OR “mediat*” OR “moderat*” OR “effect size” OR “outcomes” OR “odds”)

1 AND 2 AND 3 AND 4 595 results

Search Google Scholar (05-03-2021)

school|teacher "corporal punishment"|"physical punishment"|"physical discipline"|"physical maltreatment"|child|pupil|student|adolescent

36 selected

Section S2

Figure S1

PRISMA Flow Diagram of the Search Strategy

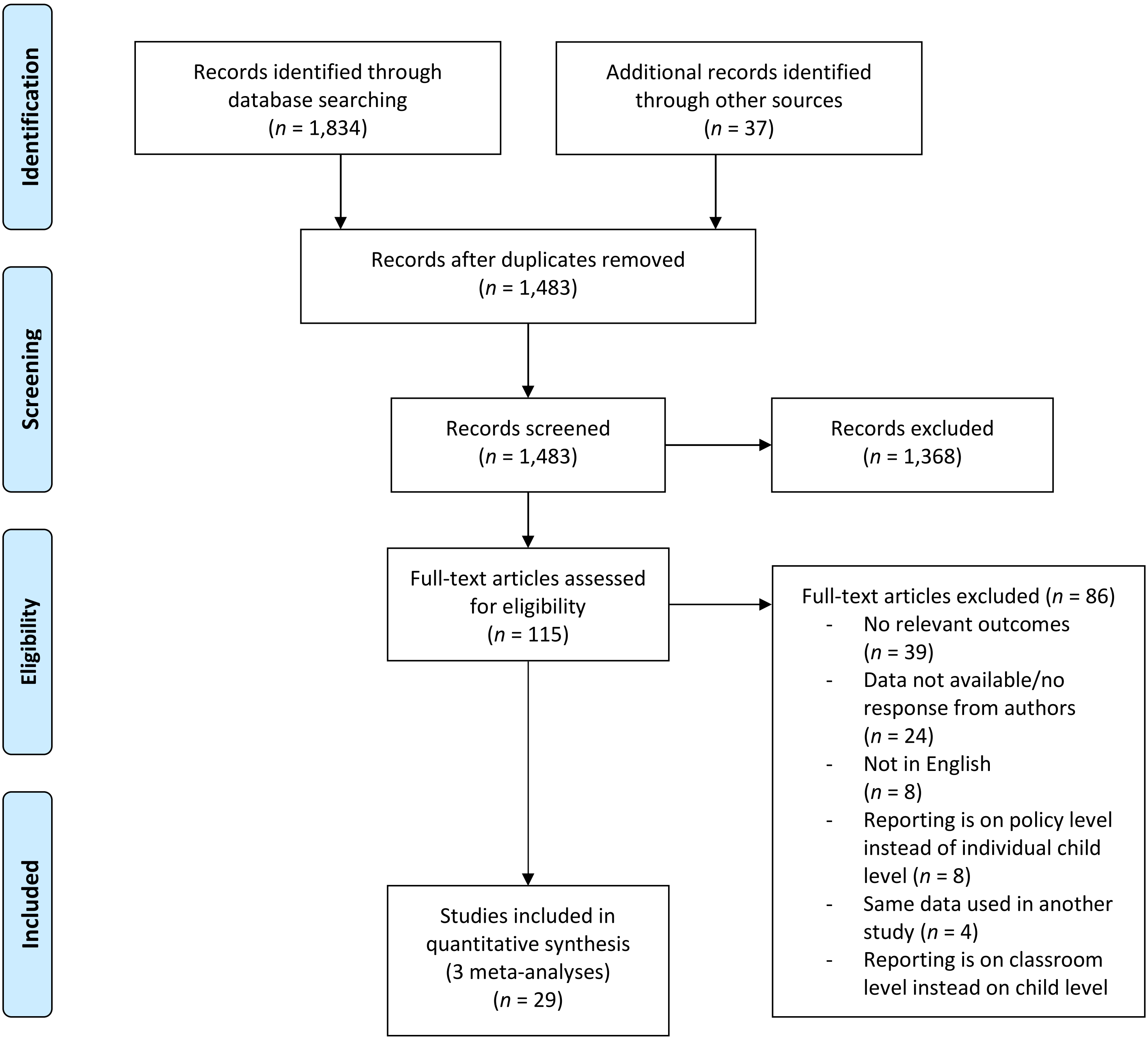

Note. The electronic databases ERIC, PsycINFO, Web of Science, and Google Scholar were searched.

Section S3

Characteristics of the Included Studies

Table S1

Characteristics of the 29 Included Studies on SCP and Externalizing Behavior Problems, Internalizing Behavior Problems, and School Performance

Author	Year	*N*	Country	Outcome(s)	Status	Study design	% Male	Age range of the children (min-max)	Mean age	# Effect sizes extracted
Ahmed et al.	2015	3,509	Malaysia	Internalizing behavior problems	P	C	51.8	10-12	NA	1
Anderson et al.	2019		United States of America	School performance	P	C				
Younger sample		564,990					51.2	NA	NA	4
Older sample		387,062					50.8	NA	NA	2
Ani & Grantham-McGregor	1998	94	Nigeria	Externalizing behavior problems	P	C	100	10-13	11.70	1
Baker-Henningham et al.	2009	1,300	Jamaica	School performance	P	C	49.0	NA	11.00	6
Benbenisthy et al.	2019			Externalizing behavior problems	P	C				
Chile		24,243	Chile				44.2	NA	NA	12
Israel		4,557	Israel				Jewish: 51.2<br>Arab: 46.8	NA	NA	48
Benbenisthy et al.	2021	601	Cameroon	Externalizing behavior problems	P	C	38.0	9-20	NA	18
Boyden	2018			School performance	U	C				
Young cohort		1,885	Ethiopia				52.8	7-11	8.12	3

Author	Year	*N*	Country	Outcome(s)	Status	Study design	% Male	Age range of the children (min-max)	Mean age	# Effect sizes extracted
Old cohort		974	Ethiopia				51.2	14-16	15.03	3
Young cohort		1,931	India				53.5	7-9	7.95	3
Old cohort		977	India				49.4	14-16	14.94	3
Young cohort		2,051	Peru				50.4	7-9	7.91	3
Old cohort		714	Peru				53.4	14-17	14.89	3
Young cohort		1,964	Vietnam				51.2	7-9	8.05	3
Old cohort		976	Vietnam				49.6	14-16	15.05	3
Bravo-Sanzana et al.	2020	2,140	Chile	School performance	P	C	41.0	14-19	15.85	1
[a]Chen & Wei	2011	1,376	Taiwan	Externalizing and internalizing behavior problems	P	C	48.2	NA	12-15	3
Chen & Wei	2013	1,650	Taiwan	Internalizing behavior problems	P	C	51.0	NA	12-15	1
Chen et al.	2020	1,262	Taiwan	Externalizing behavior problems	P	C	38.3	NA	12-15	2
Choi	2021	4,051	South Korea	Externalizing behavior problems	P	L	51.0	NA	12-15	6
Corboz et al.	2019		Afghanistan	Externalizing and internalizing behavior problems	P	C				
Girls		420					0	14.24	10-18	2
Boys		350					100	14.48	12-19	2
Csorba et al.	2001	526	Hungary	Internalizing behavior problems	P	C	67.0	11.60	NA	1
Deb et al.	2017	519	India	Internalizing behavior problems	P	C	62.6	NA	11-16	1
Devries et al.	2014		Uganda	Externalizing behavior problems	P	C				

Author	Year	*N*	Country	Outcome(s)	Status	Study design	% Male	Age range of the children (min-max)	Mean age	# Effect sizes extracted
				and school performance						
Girls		1,937					0	NA	NA	3
Boys		1,769					100	NA	NA	3
Gershoff et al.*	2019	830	United States of America	Internalizing behavior problems and school performance	P	C	45.0	21.00	18-23	2
Gundersen & McKay	2019	1,652	The Gambia	School performance	P	C	51.0	12.38	7.22	3
[b]He et al.	2019	6,576	China	Internalizing behavior problems	P	C	56.3	13.37	10-19	1
[b]He et al.	2021	6,576	China	Internalizing behavior problems	U	C	56.3	13.37	10-19	1
Hecker et al.	2014	409	Tanzania	Externalizing behavior problems	P	C	52.0	10.49	6-15	4
Khuwaja et al.	2018		Pakistan	Externalizing behavior problems, internalizing behavior problems, and school performance	P	C				
Girls		930					0	12.27	9-18	3
Boys		822					100	12.51	8-19	3
Kızıltepe et al.	2020	293	Turkey	Externalizing behavior problems, internalizing behavior problems, and school performance	P	C	50.5	12.59	10-15	4
[c]Nkuba et al.	2018	700	Tanzania	Externalizing behavior problems and internalizing behavior problems	P	C	48.0	14.92	12-17	3
[c]Nkuba et al.	2019	700	Tanzania	Externalizing behavior problems	P	C	48.0	14.92	12-17	2

Author	Year	*N*	Country	Outcome(s)	Status	Study design	% Male	Age range of the children (min-max)	Mean age	# Effect sizes extracted
Oriol et al.	2017	21,416	Peru	Externalizing behavior problems and internalizing behavior problems	P	C	53.83	13.69	13-17	3
[a]Wei et al.	2010	1,376	Taiwan	Externalizing behavior problems and internalizing behavior problems	P	C	48.2	NA	12-15	4
[d]Youssef et al.	1998	2,170	Egypt	Externalizing behavior problems and school performance	P	C	61.0	14.58	10.5-20	10
[d]Youssef et al.	1999	2,170	Egypt	Externalizing behavior problems	P	C	61.0	14.48	10.5-20	1

Note. Year = publication year; *N* = sample size; Country = country in which a study was performed; Outcome(s) = outcomes reported in a primary study; Status = published (P) or unpublished (U); Study design = cross-sectional (C) or longitudinal (L); % Male = percentage of males in a sample; Minimum and maximum age = the minimum and maximum age of a sample; Mean age = the mean age of a sample in years; Effect sizes extracted = number of effect sizes extracted from a study.
Studies sharing the same subscript ([a], [b], [c], [d]) were assigned the same study ID in the dataset as the authors of these primary studies reported on the same (sub)sample of participants. NA = Not Available, and indicates that the information could not be retrieved from the primary study.
[*] Gershoff et al. (2019) asked emerging adults to retrospectively reflect on their schooling years. Therefore, the mean age of this sample is higher than 18.
From several studies a relatively high number of effect sizes (10 or more) could be extracted as these studies reported on multiple/diverse forms of SCP. All these effect sizes met our inclusion criteria, and were this included in the review.

Section S4

References of Included Studies

Ahmed, A., Wan-Yuen, C., Marret, M. J., Guat-Sim, C., Othman, S., & Chinna, K. (2015). Child maltreatment experience among primary school children: A large scale survey in Selangor state, Malaysia. *PLOS ONE, 10(*3), Article e0119449. https://doi.org/10.1371/journal.pone.0119449

Anderson, K. P., Ritter, G. W., & Zamarro, G. (2019). Understanding a vicious cycle: The relationship between student discipline and student academic outcomes. *Educational Researcher, 48*(5), 251–262. https://doi.org/10.3102/0013189X19848720

Ani, C., & Grantham-McGregor, S. (1998). Family and personal characteristics of aggressive Nigerian boys: Differences from and similarities with Western findings. *Journal of Adolescent Health, 23*(5), 311–317. https://doi.org/10.1016/S1054-139X(98)00031-7

Baker-Henningham, H., Meeks-Gardner, J., Chang, S., & Walker, S. (2009). Experiences of violence and deficits in academic achievement among urban primary school children in Jamaica. *Child Abuse and Neglect*, *33*(5), 296–306. https://doi.org/10.1016/j.chiabu.2008.05.011

Benbenishty, R., Astor, R. A., López, V., Bilbao, M., & Ascorra, P. (2019). Victimization of teachers by students in Israel and in Chile and its relations with teachers' victimizationof students. *Aggressive Behavior, 45*(2), 107–119. https://doi.org/10.1002/ab.21791

Benbenishty, R., Daru, E. R., & Astor, R. A. (2021). An exploratory study of the prevalence and correlates of student maltreatment by teachers in Cameroon. *International Journal of Social Welfare*. Advance online publication. https://doi.org/10.1111/ijsw.12475

Boyden, J. (2018). Young Lives: An international study of childhood poverty: Round 3, 2009. [Data collection]. 3rd Edition. UK Data Service. SN: 6853. http://doi.org/10.5255/UKDA-SN-6853-3

Bravo-Sanzana, M., Salvo-Garrido, S., Miranda-Vargas, H., & Bangdiwala, S. I. (2020). What school social climate factors affect mathematics performance in secondary school students? A multilevel análisis [¿ Qué factores de Clima Social Escolar afectan el desempeño de Matemática en estudiantes secundarios? Un análisis multinivel]. *Culture and Education, 32*(3), 506-528. https://doi.org/10.1080/11356405.2020.1785138

Chen, J.-K. & Wei, H.-S. (2011). The impact of school violence on self-esteem and depression among Taiwanese junior high school students. *Social Indicators Research, 100*(3),479–498. https://doi.org/10.1007/s11205-010-9625-4

Chen, J.-K., & Wei, H.-S. (2013). School violence, social support and psychological health among Taiwanese junior high school students. *Child Abuse & Neglect, 37*(4), 252–262. https://doi.org/10.1016/j.chiabu.2013.01.001

Chen, J.-K., Wu, C., & Wei, H.-S. (2020). Personal, family, school, and community factors associated with student victimization by teachers in Taiwanese junior high schools: A multi-informant and multilevel analysis. *Child Abuse & Neglect, 99*, Article 104246. https://doi.org/10.1016/j.chiabu.2019.104246

Choi, B. (2021). Cycle of violence in schools: Longitudinal reciprocal relationship between student's aggression and teacher's use of corporal punishment. *Journal of Interpersonal Violence, 36*(3-4), 1168–1188. https://doi.org/10.1177/0886260517741627

Corboz, J., Siddiq, W., Hemat, O., Chirwa, E., & Jewkes, R. (2019). What works to prevent violence against children in Afghanistan? Findings of an interrupted time series

evaluation of a school-based peace education and community social norms change intervention in Afghanistan. *PLOS ONE, 14*(8), Article e0220614. https://doi.org/10.1371/journal.pone.0220614

Csorba, J., Rózsa, S., Vetro, A., Gadoros, J., Makra, J., Somogyi, E., Kaczvinszky, E., & Kapornay, K. (2001). Family- and school-related stresses in depressed Hungarian children. *European Psychiatry, 16*(1), 18–26. https://doi.org/10.1016/S0924-9338(00)00531-9

Deb, S., Kumar, A., Holden, G. W., & Simpson Rowe, L. (2017). School corporal punishment, family tension, and students' internalizing problems: Evidence from India. *School Psychology International*, *38*(1), 60–77. https://doi.org/10.1177/0143034316681378

Devries, K. M., Child, J. C., Allen, E., Walakira, E., Parkes, J., & Naker, D. (2014). School violence, mental health, and educational performance in Uganda. *Pediatrics, 133*(1), e129–e137. https://doi.org/10.1542/peds.2013-2007

Gershoff, E. T., Sattler, K. M. P., & Holden, G. W. (2019). School corporal punishment and its associations with achievement and adjustment. *Journal of Applied Developmental Psychology, 63*, 1–8. https://doi.org/10.1016/j.appdev.2019.05.004

Gundersen, S., & McKay, M. (2019). Reward or punishment? An examination of the relationship between teacher and parent behavior and test scores in the Gambia. *International Journal of Educational Development, 68*, 20–34. https://doi.org/10.1016/j.ijedudev.2019.03.006

He, G.-H., Strodl, E., Chen, W.-Q., Liu, F., Hayixibayi, A., & Hou, X.-Y. (2019). Interpersonal conflict, school connectedness and depressive symptoms in Chinese adolescents: Moderation effect of gender and grade level. *International Journal of*

Environmental Research and Public Health, 16(12), Article 2182. https://doi.org/10.3390/ijerph16122182

He, G.-H., Strodl, E., Chen, W.-Q., Liu, F., Hayixibayi, A., Hou, X.-Y., Yin, X.-N., Wen, G.-M., Sun, D.-L., Xian, D.-X., Wu, C.-A. (2021). Interpersonal conflict, school connectedness and anxiety in Chinese adolescents: Moderation effect of gender and grade level [Manuscript in preparation]. *School of Psychology and Counselling*, Queensland University of Technology.

Hecker, T., Hermenau, K., Isele, D., & Elbert, T. (2013). Corporal punishment and children's externalizing problems: A cross-sectional study of Tanzanian primary school aged children. *Child Abuse & Neglect, 38*(5), 884–892. https://doi.org/10.1016/j.chiabu.2013.11.007

Khuwaja, H. M. A., Karmaliani, R., McFarlane, J., Somani, R., Gulzar, S., Ali, T. S., Premani, Z. S., Chirwa, E. D., & Jewkes, R. (2018). The intersection of school corporal punishment and associated factors: Baseline results from a randomized controlled trial in Pakistan. *PLOS ONE, 13*(10), Article e0206032. https://doi.org/10.1371/journal.pone.0206032

Kızıltepe, R., Irmak, T. Y., Eslek, D., & Hecker, T. (2020). Prevalence of violence by teachers and its association to students' emotional and behavioral problems and school performance: Findings from secondary school students and teachers in Turkey. *Child Abuse & Neglect, 107*, Article 104559. https://doi.org/10.1016/j.chiabu.2020.104559

Nkuba, M., Hermenau, K., Goessmann, K., & Hecker, T. (2018). Mental health problems and their association to violence and maltreatment in a nationally representative sample of Tanzanian secondary school students. *Social Psychiatry and Psychiatric Epidemiology, 53*(7), 699–707. https://doi.org/10.1007/s00127-018-1511-4

Nkuba, M., Hermenau, K., & Hecker, T. (2019). The association of maltreatment and socially deviant behavior—Findings from a national study with adolescent students and their parents. *Mental Health & Prevention, 13*, 159–168. https://doi.org/10.1016/j.mhp.2019.01.003

Oriol, X., Miranda, R., Amutio, A., Acosta, H. C., Mendoza, M. C., & Torres-Vallejos, J. (2017).Violent relationships at the social-ecological level: A multi-mediation model to predict adolescent victimization by peers, bullying and depression in early and late adolescence. *PLOS ONE, 12*(3), Article e0174139. https://doi.org/10.1371/journal.pone.0174139

Wei, H.-S., Williams, J. H., Chen, J.-K., & Chang, H.-Y. (2010). The effects of individual characteristics, teacher practice, and school organizational factors on students' bullying: A multilevel analysis of public middle schools in Taiwan. *Children and Youth Services Review,32*(1), 137–143. https://doi.org/10.1016/j.childyouth.2009.08.004

Youssef, R. M., Attia, M. S.-E.-D., & Kamel, M. I. (1998). Children experiencing violence II: Prevalence and determinants of corporal punishment in schools. *Child Abuse and Neglect*, *22*(10), 975–985. https://doi.org/10.1016/S0145-2134(98)00084-2

Youssef, R. M., Attia, M. S.-E.-D., & Kamel, M. I. (1999). Violence among schoolchildren in Alexandria. *Eastern Mediterranean Health Journal, 5*(2), 282–298. https://www.researchgate.net/publication/12523194_Violence_among_schoolchildren_in_Alexandria

Section S5

Coding Scheme for the Included Studies

In case of missing or unknown values, leave field blank.

General Information article

1. Study ID number (1, 2, 3...): [studyID]

2. Effect size ID (1, 2, 3 …): [effectID]

3. Bibliographic reference: Write a complete citation in APA form [bib]

____

____

4. Coder name: [coder]

5. Date of coding: [cdate]

6. Year of publication: [pubyear]

7. Publication status: [pubstatus]
    1. Published (in a peer-reviewed journal)
    2. Unpublished

Sample Descriptors

8. Sample size of children on which the effect size is based: [Neff]

9. Sample size of children/adults in a sample (that reported on SCP): [Nchild]

10. Overall mean age of the sampled children in years (at first measurement): [agechild]

11. Age range of the sampled children (oldest – youngest) in years: [agerangechild]

12. Type of school: [typeschool]
    1. Primary
    2. Secondary
    3. Both

13. Percentage of children that is corporally punished at school: [SCPsample]

14. Percentage of boys in the sample: [percboy]

Research design and quality descriptors

15. Country in which a study was performed: __________ [country]

16. Continent in which a study was performed: [continent]
    1. Europe
    2. Asia

3. Africa
4. North America
5. South America
6. Other (specify): __________

17. Is the study design cross-sectional or longitudinal? [designlongcros]
    1. Cross-sectional
    2. Longitudinal

18. Instrument that was used to measure SCP: [instrumentSCP]
    1. Questionnaire
    2. Interview
    3. Observation

19. Time period in which SCP frequency and/or occurrence was measured: [durSCP]
    1. Previous day
    2. Previous week
    3. Last month/last two months
    4. Current semester/last semester
    5. Current school year
    6. Current (calendar) year
    7. Previous (calendar) year
    8. Ever
    9. In a ‘typical’ week

20. Operationalization of SCP/severity of SCP: [operSCP]
    1. Punishment with hand(s) and/or object(s)
    2. Punishment without any object
    3. Punishment only with object(s)
    4. Punishment, not further defined

21. Label for SCP used in a primary study: [labelSCP]
    1. Corporal punishment
    2. Physical violence
    3. Physical maltreatment
    4. Physical punishment
    5. Physical victimization by teacher
    6. Physical discipline
    7. Teacher’s aggressive behavior

22. Instrument used for measuring externalizing behavior problems: [instrumentext]
    1. Questionnaire
    2. Interview
    3. Observation

23. Operationalization of externalizing problems: [operext]
    1. Bullying/peer perpetration
    2. Being verbally aggressive
    3. Being physically aggressive
    4. Having conduct disorder / conduct problems

5. Being hyperactive
6. Showing delinquent and/or rule-breaking behavior
7. Total scale score on the Strengths and Difficulties questionnaire
8. Being stubborn
9. Telling lies (often)
10. Being physically and verbally aggressive

24. Instrument used for measuring internalizing behavior problems: [instrumentint]
    4. Questionnaire
    5. Interview
    6. Observation

25. Type of internalizing behavior problems: [operint]
    1. Depression
    2. Anxiety
    3. Emotional problems
    4. Low self-esteem
    5. Depression and anxiety
    6. Loneliness

26. Instrument used for measuring school performance: [instrumentcog]
    1. Questionnaire
    2. Interview
    3. Test/assessment

27. Operationalization of school performance: [opercog]
    1. Spelling skills
    2. Reading skills
    3. Vocabulary score
    4. Mathematics performance
    5. Frequency/Occurrence of repeating a school year or retaking exam(s)
    6. Grade average(s)
    7. (Aggregated) Index of language, reading, and mathematics skills

28. Dimension of SCP that is measured: [SCPindexarticle]
    1. Frequency
    2. Frequency and types
    3. Present versus absent
    4. Present versus absent and types

Effect Size Level Coding Form

29. Effect size label (describe effect size in terms of type and outcome) [eslabel]

30. Outcome of interest [outcomeES]
    1. School performance
    2. Internalizing behavior
    3. Externalizing behavior

31. Is the effect size adjusted or unadjusted? [statadj]
    1. Unadjusted
    2. Adjusted

32. Effect size (Pearson's *r*) [escorrvalue]

Section S6

Table S1. *Significant and Non-Significant Results for Categorical and Continuous Variables Tested as Moderators (in Bivariate Models).*

Variables tested as moderators	# Studies	# ES	Intercept (95% CI) / Mean *Z* (95% CI)	Mean *r*	β (95% CI)	*F*(df1, df2)[a]	*p*[b]
**Externalizing behavior problems**							
Study characteristics							
Continent in which a study was performed						*F*(2, 117) = 2.661	.074[+]
Asia (RC)	11	64	0.320 (0.260; 0.379)[***]	.310			
Africa	6	31	0.202 (0.119; 0.284)[***]	.199	-0.118 (-0.220; -0.017)[*]		
Other	4	25	0.277 (0.179; 0.374)[***]	.270	0.043 (-0.157; 0.071)		
Type of school corporal punishment						*F*(2, 117) = 3.008	.053[+]
With hand and/or objects (RC)	12	35	0.248 (0.187; 0.308)[***]	.243			
Without objects	6	72	0.333 (0.267; 0.399)[***]	.321	0.085 (-0.005; 0.175)[+]		
Only with objects or not defined	4	13	0.267 (0.191; 0.344)[***]	.261	0.020 (-0.078; 0.117)		
Method used for measuring SCP						*F*(1, 118) = 0.307	.581
Questionnaire (RC)	15	109	0.286 (0.233; 0.339)[***]	.278			
Interview	6	11	0.256 (0.163; 0.349)[***]	.251	-0.030 (-0.137; 0.077)		
Time period in which SCP was measured						*F*(2, 113) = 2.790	.066[+]
Last week, month or two months (RC)	14	89	0.306 (0.255; 0.357)[***]	.297			
From this semester to last year	5	23	0.246 (0.164; 0.328)[***]	.241	-0.062 (-0.157; -0.037)		
Ever	1	4	0.098 (-0.085; 0.281)	.098	-0.208 (-0.398; -0.018)[*]		
Method for measuring externalizing behavior						*F*(1, 118) = 0.014	.906
Questionnaire (RC)	16	110	0.280 (0.228; 0.333)[***]	.273			
Interview	5	10	0.274 (0.175; 0.372)[***]	.267	-0.007 (-0.119; 0.105)		
Dimension of SCP that was measured						*F*(2, 117) = 5.058	.008[**]
Frequency (RC)	8	80	0.314 (0.258; 0.369)[***]	.304			
Present versus absent	7	22	0.191 (0.126; 0.256)[***]	.189	-0.123 (-0.208; -0.037)[**]		
Frequency and types	6	18	0.321 (0.251; 0.390)[***]	.310	0.007 (-0.082; 0.096)		
Operationalization of externalizing behavior						*F*(9, 110) = 2.600	.009[**]
Delinquency/rule-breaking behavior (RC)	11	36	0.267 (0.217; 0.318)[***]	.261			
Being physically aggressive	11	31	0.293 (0.241; 0.345)[***]	.285	0.026 (-0.006; 0.058)		
Being verbally aggressive	8	28	0.261 (0.208; 0.315)[***]	.256	-0.006 (-0.039; 0.027)		
Peer perpetration/bullying	7	7	0.375 (0.303; 0.447)[***]	.358	0.108 (0.028; 0.187)[**]		
Being physically and verbally aggressive	1	6	0.300 (0.136; 0.465)[***]	.291	0.033 (-0.139; 0.205)		

Variables tested as moderators	# Studies	# ES	Intercept (95% CI) / Mean *Z* (95% CI)	Mean *r*	β (95% CI)	*F*(df1, df2)[a]	*p*[b]
Total scale score on SDQ	2	4	0.178 (0.050; 0.305)**	.176	-0.089 (-0.226; 0.048)		
Having conduct disorder/conduct problems	3	3	0.192 (0.083; 0.300)***	.189	-0.075 (-0.184; 0.033)		
Being hyperactive	3	3	0.167 (0.058; 0.275)**	.165	-0.101 (-0.209; 0.007)		
(Often) Telling lies	1	1	0.236 (0.098; 0.374)**	.232	-0.031 (-0.161; 0.100)		
Being stubborn	1	1	0.149 (0.011; 0.287)*	.148	-0.118 (-0.248; 0.013)		
Sample characteristics							
Mean age of the sample in years	10	26	0.275 (0.163; 0.387)***	-	0.007 (-0.071; 0.085)	*F*(1, 24) = 0.034	.855
Percentage of boys in the sample	21	120	0.279 (0.233; 0.326)***	-	-0.000 (-0.002; 0.002)	*F*(1, 118) = 0.072	.790
Type of school						*F*(2, 117) = 0.151	.860
Both (RC)	7	88	0.290 (0.214; 0.366)***	.282			
Secondary school	8	21	0.282 (0.203; 0.361)***	.274	-0.009 (-0.118; 0.101)		
Primary school	6	11	0.257 (0.161; 0.352)***	.251	-0.033 (-0.156; 0.089)		
**Internalizing behavior problems**							
Study characteristics							
Continent in which a study was performed						*F*(1, 16) = 0.182	.676
Asia (RC)	9	12	0.163 (0.115; 0.212)***	.162			
Other	5	6	0.146 (0.077; 0.216)***	.145	-0.017 (-0.102; 0.068)		
Type of school corporal punishment						*F*(1, 13) = 0.001	.983
With hand and/or objects (RC)	9	10	0.154 (0.101; 0.208)***	.153			
Not defined	3	5	0.155 (0.081; 0.229)***	.154	0.001 (-0.090; 0.092)		
Time period in which SCP was measured						*F*(2, 12) = 3.484	.064+
Last month (RC)	5	5	0.218 (0.147; 0.289)***	.215			
From last semester to previous year	6	8	0.147 (0.097; 0.197)***	.146	-0.071 (-0.158; 0.016)		
Ever	2	2	0.076 (-0.022; 0.173)	.076	-0.142 (-0.263; -0.022)*		
Dimension of SCP that was measured						*F*(2, 15) = 1.208	.326
Present versus absent (RC)	6	7	0.146 (0.084; 0.207)***	.145			
Frequency and types	5	7	0.191 (0.129; 0.253)***	.189	0.046 (-0.042; 0.133)		
Frequency	3	4	0.123 (0.046; 0.200)**	.122	-0.023 (-0.121; 0.076)		
Operationalization of internalizing behavior						*F*(5, 12) = 0.885	.521
Being depressed (RC)	11	12	0.173 (0.120; 0.226)***	.171			
Having emotional problems	2	2	0.159 (0.020; 0.298)*	.158	-0.014 (-0.162; 0.135)		
Being anxious	1	1	0.109 (-0.049; 0.266)	.108	-0.065 (-0.225; 0.096)		
Being lonely	1	1	0.139 (-0.016; 0.294)	.138	-0.034 (-0.192; 0.123)		
Having low(er) self-esteem	1	1	0.032 (-0.130; 0.195)	.032	-0.141 (-0.303; 0.022)		
Being both depressed and anxious	1	1	0.182 (-0.009; 0.373)	.180	0.009 (-0.190; 0.208)		
Statistical adjustment of effect sizes						*F*(1, 16) = 0.398	.537

Variables tested as moderators	# Studies	# ES	Intercept (95% CI) / Mean *Z* (95% CI)	Mean *r*	β (95% CI)	*F*(df1, df2)[a]	*p*[b]
Unadjusted (RC)	12	16	0.153 (0.111 0.196)***	.152			
Adjusted	2	2	0.191 (0.072; 0.310)**	.189	0.038 (-0.089; 0.164)		
Sample characteristics							
Mean age of the sample in years	10	12	0.179 (0.129; 0.229)***	-	-0.021 (-0.041; -0.002)*	*F*(1, 10) = 5.773	.037*
Percentage of boys in the sample	14	18	0.157 (0.118; 0.197)***	-	-0.001 (-0.002; 0.001)	*F*(1, 16) = 0.385	.544
Type of school						*F*(2, 15) = 2.889	.087+
Secondary school (RC)	8	11	0.150 (0.104; 0.197)***	.149			
Primary school	4	4	0.223 (0.146; 0.299)***	.219	0.073 (-0.017; 0.162)		
Both	2	3	0.098 (0.014; 0.181)*	.097	-0.053 (-0.148; 0.043)		
**School performance**							
Study characteristics							
Continent in which a study was performed						*F*(3, 43) = 1.326	.278
Asia (RC)	6	14	-0.103 (-0.178; -0.028)**	-.103			
North America	4	13	-0.106 (-0.196; -0.015)*	-.106	-0.003 (-0.120; 0.115)		
Africa	5	11	-0.060 (-0.141; 0.021)	-.060	0.043 (-0.068; 0.153)		
Other	5	9	-0.174 (-0.258; -0.091)***	.172	-0.071 (-0.184; 0.041)		
Method used for measuring SCP						*F*(1, 39) = 3.505	.069+
Interview (RC)	14	37	-0.101 (-0.147; -0.054)***	-.100			
Questionnaire	4	4	-0.196 (-0.287; -0.104)***	-.193	-0.095 (-0.198; 0.008)+		
Time period in which SCP was measured						*F*(4, 42) = 1.461	.231
Previous day or week (RC)	9	23	-0.066 (-0.126; -0.007)*	-.066			
Current school year to previous school year	5	14	-0.133 (-0.212; -0.054)**	-.132	-0.067 (-0.166; 0.032)		
Last month	3	3	-0.208 (-0.320; 0.096)***	-.205	-0.142 (-0.269; -0.015)*		
In a ‘typical’ school week	2	6	-0.104 (-0.230; 0.022)	-.104	-0.038 (-0.178; 0.101)		
Ever	1	1	-0.148 (-0.336; 0.040)	-.147	-0.082 (-0.279; 0.115)		
Dimension of SCP that was measured						*F*(1, 45) = 0.298	.588
Present versus absent and types (RC)	5	12	-0.130 (-0.214; -0.046)**	-.129			
Frequency and types	15	35	-0.103 (-0.152; -0.054)***	-.103	-0.026 (-0.071; 0.124)		
Operationalization of school performance						*F*(6, 40) = 1.823	.119
Mathematics performance (RC)	11	13	-0.089 (-0.139; -0.039)***	-.089			
Reading skills	10	13	-0.103 (-0.154; -0.052)***	-.103	-0.014 (-0.040; 0.012)		
Vocabulary score	8	8	-0.072 (-0.124; -0.019)**	-.071	0.017 (-0.012; 0.047)		
Grade average(s)	4	4	-0.192 (-0.290; -0.095)***	-.190	-0.103 (-0.213; 0.006)		
(Aggregated) Index of language, reading, and mathematics skills	3	4	-0.094 (-0.144; -0.044)***	-.093	-0.005 (-0.013; 0.004)		
Frequency/Occurrence of repeating a school year or retaking exam(s)	2	3	-0.106 (-0.233; 0.021)	-.106	-0.017 (-0.153; 0.119)		

Variables tested as moderators	# Studies	# ES	Intercept (95% CI) / Mean *Z* (95% CI)	Mean *r*	β (95% CI)	*F*(df1, df2)[a]	*p*[b]
Spelling skills	1	2	-0.066 (-0.147; 0.015)	-.066	0.023 (-0.045; 0.090)		
Publication status of primary study						*F*(1, 45) = 2.083	.156
Unpublished (RC)	8	24	-0.075 (-0.138; -0.011)*	-.075			
Published	12	23	-0.134 (-0.187; -0.081)***	-.133	-0.059 (-0.142; 0.024)		
Sample characteristics							
Mean age of the sample in years	16	39	-0.124 (-0.163; -0.089)***	-	-0.009 (-0.020; 0.002)	*F*(1, 37) = 2.701	.109
Percentage of boys in the sample	20	47	-0.110 (-0.151; -0.069)***	-	-0.001 (-0.001; 0.003)	*F*(1, 45) = 1.423	.239
Type of school						*F*(2, 44) = 0.262	.771
Primary school (RC)	10	25	-0.099 (-0.160; -0.037)**	-.098			
Both	7	18	-0.113 (-0.186; -0.039)**	-.112	0.014 (-0.110; 0.082)		
Secondary school	3	4	-0.145 (-0.261; -0.029)*	-.144	-0.047 (-0.178; 0.084)		

Note. # Studies = number of independent study samples; # ES = number of effect sizes; Mean *Z* = Mean effect size (Fisher's *Z*); CI = confidence interval; Mean *r* = Mean effect size (Pearson's correlation); β = estimated regression coefficient; RC = Reference category; SDQ = Strengths and Difficulties Questionnaire.

[a] Omnibus test of all regression coefficients in the model.

[b] *p*-value of the omnibus test.

$^{+}p < .10$; $^{*}p < .05$; $^{**}p < .01$; $^{***}p < .001$.

Section S7

Funnel Plots

Figure S1

Funnel Plot for the Externalizing Behavior Meta-Analysis

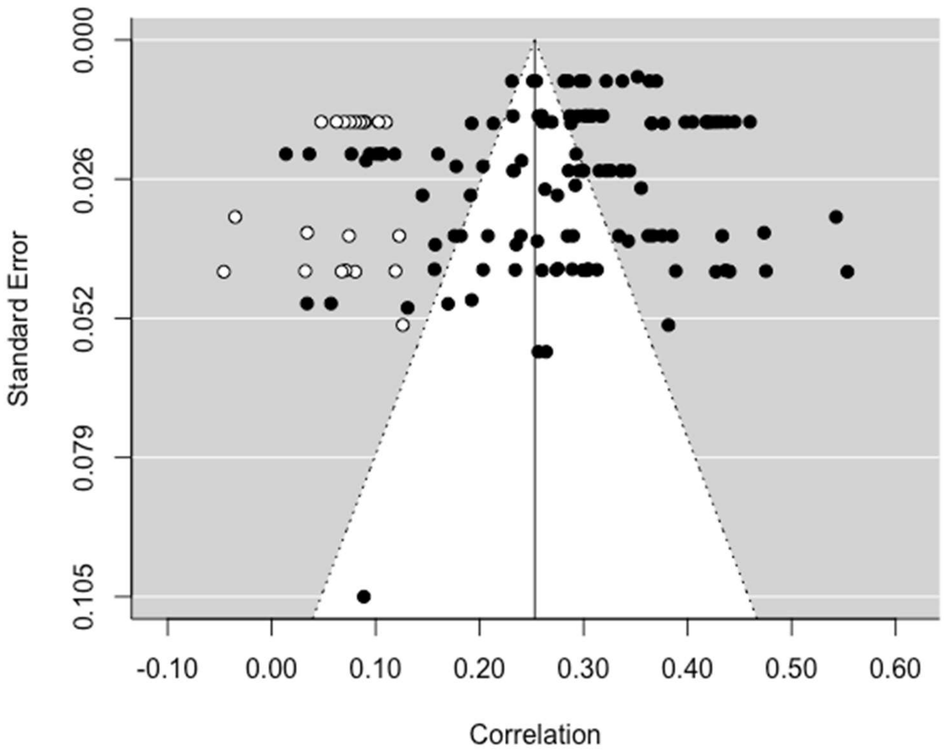

Note. The white dots illustrate that 21 effect sizes were missing on the left side, indicating that the estimated mean effect may be an overestimation of the true effect. This implies that the results of meta-analysis on externalizing behavior problems may have been affected by publication bias.

Figure S2

Funnel Plot for the Internalizing Behavior Meta-Analysis

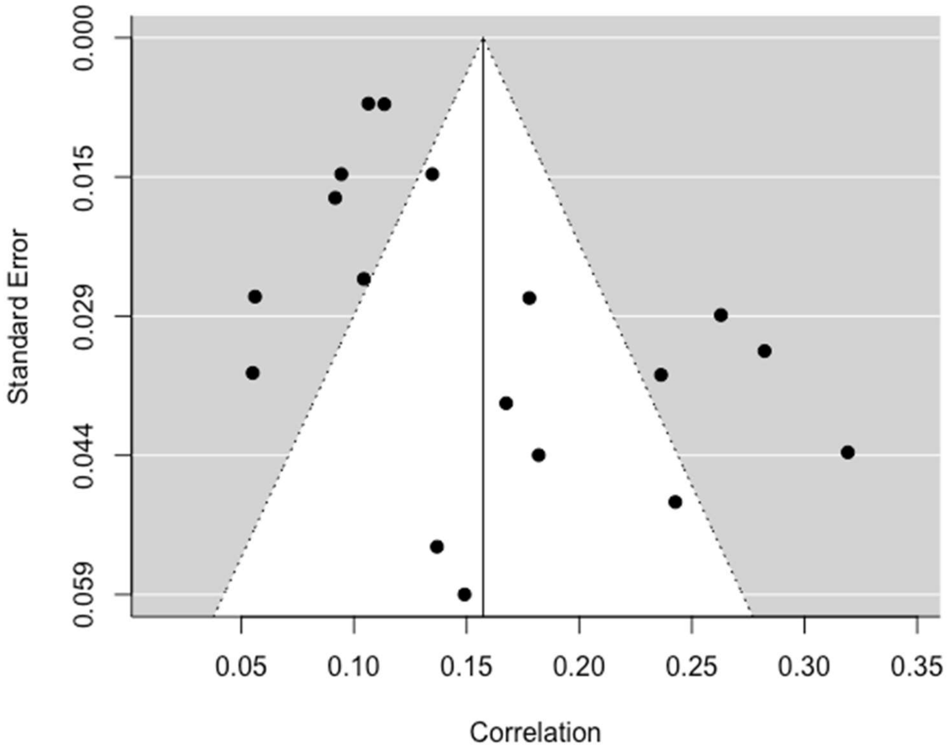

Note. No effect sizes had to be imputed according to the trim-and-fill algorithm, implying there were no indications for publication bias.

Figure S3

Funnel Plot for the School Performance Meta-Analysis

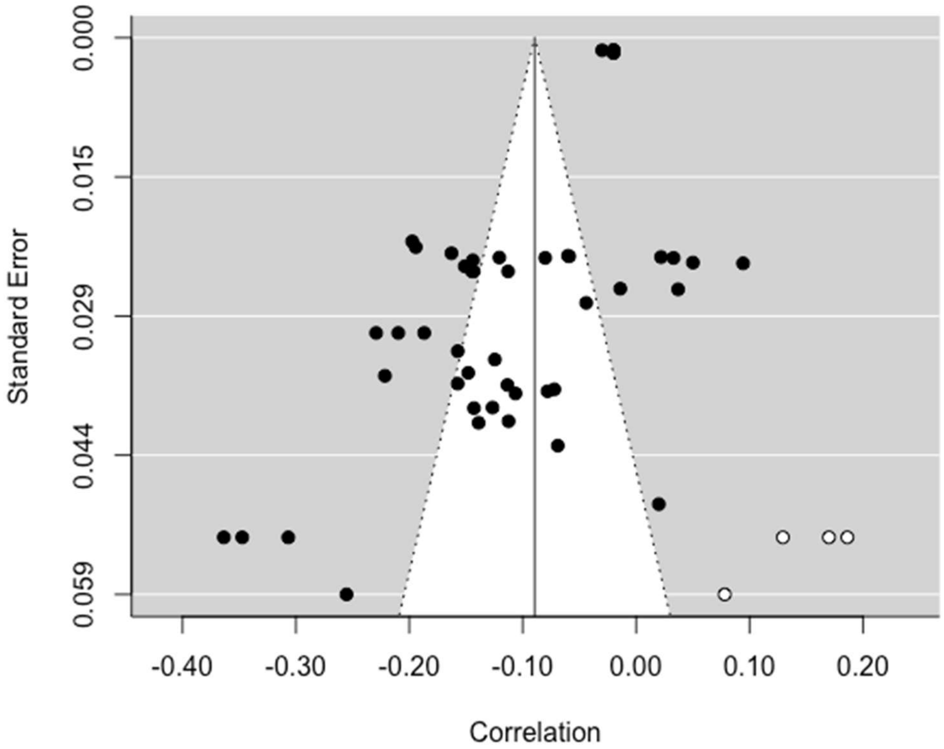

Note. The white dots illustrate that 4 effect sizes were imputed on the right side to restore the symmetry of the plot, indicating that the estimated mean effect may be an underestimation of the true effect. These results reveal that the results may have been affected by (selection) bias.
